# Symptoms of Anxiety and Depression within the UNiversity community: the cross-sectional UN-SAD study

**DOI:** 10.1186/s12889-022-13876-5

**Published:** 2022-08-03

**Authors:** E. Scarpis, M. Del Pin, E. Ruscio, A. Tullio, S. Brusaferro, L. Brunelli

**Affiliations:** 1 Dipartimento di Area Medica, Università degli studi di Udine, Via Colugna 50, 33100 Udine, Italy; 2 Istituto di Igiene ed Epidemiologia Clinica, Azienda Sanitaria Universitaria Friuli Centrale, Udine, Italia; 3 Rischio Clinico, Qualità e Accreditamento, Azienda Sanitaria Universitaria Friuli Centrale Udine, Italia

**Keywords:** Mental health, University, Anxiety, Depression, Career

## Abstract

The increasing complexity of academia, with its demanding working conditions and uncertain career opportunities, may affect the mental health of academics and potentially lead to mental health problems. The aim of this study is to determine the prevalence of depressive and anxiety symptoms in the academic population of the University of Udine and to compare symptoms in senior and younger academics and administrative staff.

A cross-sectional survey was conducted between June and December 2020, involving academic and administrative staff in all departments. The prevalence of depressive and anxiety symptoms was assessed using the PHQ-9 and GAD-7 tools. The relationship between mental health outcomes and job role was analyzed using nonparametric tests and ordinal logistic regression.

A total of 366 individuals participated: 109 junior academics, 146 senior and 111 administrative staff. The proportion of women was 55.7% and the mean age was 47.9 years. The prevalence of depressive and anxiety symptoms in the studied population was 25.7% (95% IC 21.5–30.4) and 22.7% (95% IC 18.7–27.2) respectively, with junior academics having the higher prevalence of both symptoms. Univariate models suggest a higher risk for anxiety symptoms OR 1.89 (1.13–3.17) for women.

The prevalence of depressive symptoms is higher in our academic community than in the general population, especially among junior academics. These findings may reflect the impact of uncertain career and challenging environment on the mental health of young academics. Universities should provide more support to young academics so that they can contribute effectively and healthily to the advancement of research.

## Introduction

The academic profession is in some ways atypical: professionals are intrinsically motivated and in most cases experience high levels of job satisfaction, but they can suffer from high levels of external pressure, leading to psychological problems [1]. Recently, many concerns have been raised about the potential impact of working conditions and research and career opportunities on the mental health of academics [2–4]. Indeed, the complexity of the academia has increased, in recent years, as more attention has been paid to accountability, resource management and the internationalization of research [5–7]. All these changes have affected traditional academic profiles, which are mainly characterized by the key role of teaching, research, and institutional mission [8]. Recent studies have shown that despite the remaining positive elements that characterize the academic profession, the main causes of stress are the increase in bureaucratic procedures, the number of students, and the professionals competing for reduced funding opportunities [9]. Some authors noted that the “publish or perish” imperative very often has a counterproductive effect and puts enormous pressure on the publication of research results, leading to increased stress levels and lower job satisfaction, especially among younger academics and women [10–13]. Increased workload, reduced autonomy and salary, overuse of fixed-term contracts, and resulting job insecurity, along with lack of promotion opportunities and difficulty balancing work life, can have a significant impact on the mental health of academics, especially younger academics [2–4, 13–16]. Moreover, some previous research suggests greater vulnerability among women who experience high levels of family and work stress and increased pressure to publish scientific papers, leading them to consider leaving their jobs [17, 18].

Despite these conditions, there is still a lack of knowledge about the current burden of mental illness in the academic community. Recent studies looking at anxiety and depressive symptoms in medical students and physicians during their residency program found a prevalence of mental health problems ranging from 20.9 to 43.2% [19–21]. These data are much higher than the prevalence in the general population, which is 4.6–9.3% [22, 23]. A 2003 study by Winefield et al. conducted at 17 Australian universities provides preliminary evidence of the high levels of stress experienced by academic professionals: 43% of them suffered from some degree of psychological distress. Their findings suggest that academics are more affected by mental health problems compared to technical and administrative staff at the same university [24].

An accurate assessment of the prevalence of anxiety and depressive symptoms representative of possible mental disorders in academics is the first step in providing prevention and support strategies for university members to prevent *burnout* and improve psychosocial functioning. For this reason, we decided to investigate the presence and extent of anxiety and depressive symptoms among academic professionals at the University of Udine (Italy). The aims of our study are to 1) determine the prevalence of anxiety and depressive symptoms; 2) compare the prevalence between senior, junior and administrative staff; 3) determine the demographic characteristics of those with higher prevalence.

## Methods

### Study design and setting

Between June and December 2020, we conducted a cross-sectional study (UN-SAD: Symptoms of Anxiety and Depression within the UNiversity community) at the University of Udine, a university in northeastern Italy with approximately 15,000 students.

### Participants

The study included all academic and administrative staff of all academic departments (Business and Economics, Life Sciences and Medicine, Basic Sciences and Engineering, Humanities, Political Sciences) except visiting professors.

### Recruitment and data collection

Data were collected using an anonymous online survey with 69 questions. Invitations to participate were sent to institutional email addresses with a request to complete the survey; reminders were sent shortly before the deadline. Participant consent was implied by completion of the questionnaire.

### Measures

The survey included questions on sociodemographic characteristics (age, sex, occupational profile, educational level, academic department, marital status, years of working experience, and commuting distance) and two validated tests of psychological assessment: the Patient Health Questionnaire–9 (PHQ-9) [25] for depressive symptoms and the General Anxiety Disorder–7 (GAD-7) [26] for anxiety symptoms. We chose these two tests because they have good sensitivity and specificity for the presence of symptoms [25, 26]. Moreover, they are widely used in both psychiatric and general medical practise to detect the presence of initial depressive and anxiety symptoms, especially in outpatient settings and in research field, thus ensuring cross-cultural applicability and comparison with previous literature.

The sample size for each group (senior, junior academics, administrative staff) was calculated with a confidence level of 95% and a precision of 7%. Based on our hypothesis of different prevalence of depression and anxiety symptoms in the three groups (15, 20, and 10% for senior academics, junior academics and administrative staff respectively), the calculated sample size was at least 86, 101, and 62 respondents for each group, respectively. The procedures performed in this study with human participants conformed to ethical standards, the 1964 Declaration of Helsinki and its subsequent amendments, or comparable ethical standards. The study was approved by the Institutional Review Board of the University of Udine, Italy.

### Data analysis

Data were reported as frequencies and percentages for categorical variables and as means and standard deviations for continuous variables. Results were presented as both categorical (PHQ-9: minimal or none, mild, moderate, moderately severe, and severe symptoms; GAD-7: none, mild, moderate, and severe symptoms) and dichotomous variables, with PHQ-9 and GAD-7 scores ≥ 10 showing high sensitivity and specificity, respectively, for detecting a diagnosis of the respective disorder compared with diagnostic tests [27–29]. Chi-square and Fisher’s Exact tests were applied to evaluate the possible association between categorised variables. The association between categorised variables and dichotomous outcomes was assessed using univariate and multivariate logistic regression analyses. The significance level was set at 0.05. All statistical analyses were performed using R. software, version 3.4.2 (R Foundation for Statistical Computing, Vienna, Austria) [R: The R Project for Statistical Computing. Available at: https://www.r-project.org/. [Last accessed on 2021 Oct 05]].

## Results

A total of 1,550 participants were eligible (600 senior academics, 500 junior academics and 450 administrative staff personnel). Of them, 366 participated in the survey, for a response rate of 23.6% (366/1550). Of these, 146 (39.9%) were senior academics, 109 (29.8%) were junior academics and 111 (30.3%) were administrative staff. In the groups of junior academics and administrative staff, most respondents were female (204; 55.7%), while the senior academics group was predominantly male (86; 58.9%). The mean age of all respondents was 47.9 ± 12.0 years, with a lower age for junior academics (33.2 ± 6.4 years). The most represented departments were Basic Sciences and Engineering (150; 41.0%) and Humanities (41; 11.2%). The full list of sociodemographic variables is shown in Table 1.

**Table 1:** Sociodemographic characteristics of respondents

	**Senior academics** **(***N* **= 146)**	**Junior academics** **(*N* = 109)**	**Administrative staff** **(*N* = 111)**	**Overall** **(*N* = 366)**
**Sex,** n (%)				
Female	60 (41.1)	58 (53.2)	86 (77.5)	204 (55.7)
Male	86 (58.9)	51 (46.8)	25 (22.5)	162 (44.3)
**Age (yr),** mean ± SD	55.6 ± 7.0	33.2 ± 6.4	52.3 ± 7.7	47.9 ± 12.0
Marital status, n (%)				
Single	15 (10.3)	67 (61.5)	16 (14.4)	98 (26.8)
Divorced/separated	13 (8.9)	1 (0.9)	13 (11.7)	27 (7.4)
Married/cohabiting couples	116 (79.5)	41 (37.6)	78 (70.3)	235 (64.2)
Widowed	2 (1.4)	0 (0.0)	4 (3.6)	6 (1.6)
Educational level, n (%)				
PhD	105 (71.9)	69 (63.3)	6 (5.4)	180 (49.2)
Medical specialty	3 (2.1)	1 (0.9)	0 (0.0)	4 (1.1)
Doctor or equivalent	3 (2.1)	0 (0.0)	6 (5.4)	9 (2.5)
Master’s or equivalent	35 (24.0)	39 (35.8)	54 (48.6)	128 (35.0)
Bachelor’s or equivalent	0 (0.0)	0 (0.0)	7 (6.3)	7 (1.9)
Upper secondary education	0 (0.0)	0 (0.0)	38 (34.2)	38 (10.4)
Profile, n (%)				
Full professor	39 (26.7)	/	/	39 (10.7)
Associate professor	70 (47.9)	/	/	70 (19.1)
Senior researcher	37 (25.3)	/	/	37 (10.1)
Junior researcher	/	22 (20.2)	/	22 (6.0)
Fellow	/	55 (50.5)	/	55 (15.0)
PhD student	/	32 (29.4)	/	32 (8.7)
Department, n (%)				
Business and Economics	22 (15.1)	8 (7.3)	/	30 (8.2)
Life Sciences and Medicine	14 (9.6)	15 (13.8)	/	29 (7.9)
Basic Sciences and Engineering	84 (57.5)	66 (60.6)	/	150 (41.0)
Humanities	23 (15.8)	18 (16.5)	/	41 (11.2)
Political Sciences	2 (1.4)	1 (0.9)	/	3 (0.8)
Missing	1 (0.7)	1 (0.9)	/	111 (30.3)
**Years of working experience (yr),** mean ± SD	25.0 ± 8.1	5.72 ± 5.1	24.7 ± 8.9	19.2 ± 11.6
**Commuting distance (km),** mean ± SD	29.7 ± 60.5	47.9 ± 112.0	15.6 ± 16.8	30.9 ± 73.5

The prevalence of depressive and anxiety symptoms in the studied population was 25.7% (95% IC 21.5–30.4) and 22.7% (95% IC 18.7–27.2), respectively (dichotomous scoring criteria). Junior academics reported higher rates of depressive (39.4%) and anxiety (33.0%) symptoms than senior academics (depressive symptoms 14.4% and anxiety symptoms 15.1%) and administrative staff (depressive symptoms 27.0% and anxiety symptoms 22.5%), using dichotomous scoring criteria. Moreover, the severity of both mental disorders was higher in junior academics than in the other two groups (*p* < 0.001). Table 2 shows the PHD-9 and GAD-7 scores, and the risk of depressive and anxiety symptoms for all groups in the academic community.

**Table 2:** Results of PHQ-9 and GAD-7 tests

	**Senior academics** **(*N*** **= 146)**	**Junior academics** **(*N* = 109)**	**Administrative staff** **(*N*** **= 111)**	Overall **(*N*** **= 366)**	***p*-value**
**PHQ-9, n (%)**					
None	62 (42.5)	26 (23.9)	38 (34.2)	126 (34.4)	< 0.001
Mild	63 (43.2)	40 (36.7)	43 (38.7)	146 (39.9)	
Moderate	14 (9.6)	31 (28.4)	24 (21.6)	69 (18.9)	
Moderately severe	7 (4.8)	7 (6.4)	6 (5.4)	20 (5.5)	
Severe	0 (0.0)	5 (4.6)	0 (0.0)	5 (1.4)	
**Depressive symptoms, n (%; 95%CI)**					
Low-risk	125 (85.6; 79.0–91.4)	66 (60.6; 51.2–69.2)	81 (73.0; 64.1–80.4)	272 (74.3; 69.6–78.5)	< 0.001
High-risk	21 (14.4; 9.6–21.0)	43 (39.4; 30.8–48.8)	30 (27.0; 19.6–35.9)	94 (25.7; 21.5–30.4)	
**GAD-7, n (%)**					
None	73 (50.0)	34 (31.2)	40 (36.0)	147 (40.2)	< 0.001
Mild	51 (34.9)	39 (35.8)	46 (41.4)	136 (37.2)	
Moderate	17 (11.6)	23 (21.1)	20 (18.0)	60 (16.4)	
Severe	5 (3.4)	13 (11.9)	5 (4.5)	23 (6.3)	
**Anxiety symptoms, n (%; 95%CI)**					
Low-risk	124 (84.9; 78.2–89.8)	73 (67.0; 57.7–75.1)	86 (77.5; 68.9–84.3)	283 (77.3; 72.8–81.3)	0.003
High-risk	22 (15.1; 10.2–21.8)	36 (33.0; 24.9–42.3)	25 (22.5; 15.7–31.1)	83 (22.7; 18.7–27.2)	

In univariate analysis, junior academics were more likely to suffer from depressive and anxiety symptoms OR (95% CI) 3.86 (2.12–7.14) and 2.77 (1.52–5.13), respectively, compared with senior academics; similar results, although less severe, were found for administrative staff, who reported a higher frequency of depressive symptoms 2.20 (1.18–4.15) and anxiety symptoms 1.64 (0.86–3.12) compared with senior academics. In our population, female sex was associated with a significantly higher risk for anxiety symptoms OR 1.89 (1.13-3.17), whereas no statistically significant differences were found for depressive symptoms OR 1.48 (0.91–2.39).

Univariate regression confirmed that administrative staff also had a higher risk for depressive symptoms OR 2.20 (1.18–4.15), but not for anxiety symptoms OR 1.64 (0.86–3.12). However, when both sex and age were taken into account (with mean age as the cutoff), the significance of the role association decreased with a relative risk for depressive symptoms of 1.92 for junior academics (0.86–4.41) and 1.91 (0.99–3.71) for administrative staff. This model for depressive symptoms showed no difference with respect to sex (*p* = 0.33), but the overall risk for depression decreased with age OR 0.43 (0.21–0.88). For anxiety symptoms, the risk was higher among junior academics OR 2.12 (0.92–5.28) and among administrative staff OR 1.30 (0.70–2.55) than among senior academics. The risk for anxiety symptoms was significantly higher in women than in men (OR 1.85 (1.07–3.19)), while there was no significant association with age (*p* = 0.54). No significant differences were found in any of the multivariate models with respect to years of work experience, commuting distance, educational profile, marital status, or university department, so these results were not presented. The results of the multivariate model are presented in Table 3.

**Table 3:** Crude and adjusted odds ratios resulting from the multivariate models for the prevalence of depressive and anxiety symptoms

Variable	Crude OR (95%CI)		Adjusted OR (95%CI)		*p*-value	
Category	PHQ-9	GAD-7	PHQ-9	GAD-7	PHQ-9	GAD-7
Junior Academics vs Senior Academics	3.86 (2.12–7.14)	2.77 (1.52–5.13)	1.92 (0.86–4.41)	2.12 (0.92–5.28)	0.114	0.083
Administrative Staff vs Senior Academics	2.20 (1.18–4.15)	1.64 (0.86–3.12)	1.91 (0.99–3.71)	1.30 (0.70–2.55)	0.055	0.435
Male vs Female	1.48 (0.91–2.39)	1.89 (1.13–3.17)	1.30 (0.77–2.19)	1.85 (1.07–3.19)	0.33	0.027
> 47.9 years vs ≤ 47.9 years	0.33 (0.20–0.54)	0.76 (0.50–1.16)	0.43 (0.21–0.88)	0.79 (0.37–1.74)	0.019	0.541

## Discussion

The UN-SAD study aimed to determine the prevalence of anxiety and depressive symptoms among academics at the University of Udine, to obtain data on mental health problems in academia [30, 31].

The results on depressive symptoms revealed a prevalence of 25.7% in our academic community, which seems to confirm a higher prevalence of moderate or severe depressive symptoms among academics compared to the general population, as reported both nationally [21] and internationally [7, 22, 23, 32]. The prevalence of depressive symptoms among junior academics (39.4%) is higher than among PhD students in biomedical sciences (10.1%) and in economics (18.0%), as well as among colleagues pursuing a master’s degree (39.0%) [30, 33, 34]. In addition, the distribution of PHQ-9 scores showed that a high proportion of junior academics suffered from moderate (28.4%), severe or moderately severe (11%) depressive symptoms. As for anxiety symptoms, only 22.7% of participants reported suffering from this mental health problem. Studies conducted in other countries such as Australia or the United Kingdom (UK) using the General Health Questionnaire (GHQ) to assess mental illness found that the prevalence of mental disorders in Australia was 43.7% [35], whereas in the UK it ranged from 31.8% among lecturers and senior lecturers [36] to 53% among academic staff [37]. It is likely that differences in the tool used for this assessment may have prevented us from making a fair comparison across data.

This study supports previous findings suggesting that the academic community is at higher risk for mental illness, particularly junior academics, compared with the general population and other occupations as reported by Winefield, e.g., engineers, transportation workers, general university staff [35]. Although some authors found no significant difference according to position or age [38, 39] and others found higher stress levels among senior positions [40], several studies reported higher stress levels among teachers in junior positions [6, 41, 42]. Some authors explained this by lower autonomy, lower salary, and greater job insecurity [1, 6, 13, 43]. In contrast to reports on Italian medical students [44], a higher prevalence of depressive or anxiety symptoms was not found among members of the academic community working away from home. Although some studies have not found an association between depressive or anxiety symptoms and sex or age [24, 31], our findings are consistent with those reporting higher levels of stress among women in academia, confirming the presence of some known gender differences in mental health problems [45, 46] even within the academic community, where women experience high levels of family and work stress [47]. In addition, depressive and anxiety symptoms occurred more frequently in our younger academics, confirming the observation of their higher levels of stress, which may be related to job insecurity [39]. In contrast to the reports of Winefield et al. [24], senior academics were less likely to suffer from depressive and anxiety symptoms than junior academics and administrative staff.

### Limitations and strengths

The present study has several limitations. The first is the use of a cross-sectional design at a single Italian university, which limits the representativeness of the study for the entire academic community. For this reason, our results should be interpreted with caution given the small sample size. Second, the choice of a direct e-mail invitation as recruitment method and the use of a self-report tool may have led to some self-selection bias, a well-known limitation of online surveys. Because the questionnaire addressed anxiety and depressive symptoms, it may have attracted participants who were more exposed to these symptoms than the rest of the group, possibly leading to an overrepresentation of depressive and anxiety symptoms. Finally, the unequal participation of the groups may have compromised the effectiveness of the comparison of occupational profiles.

Despite these limitations, this study also has some strengths. To our knowledge, this is the first Italian study that aims to assess the burden of mental health problems within the academic community by providing details on the different roles within the same university at a given time. Second, the use of two validated and widely used tools to assess depressive and anxiety symptoms (PHQ-9 and GAD-7, respectively) underpins the findings of this study, which may be useful in the implementation of prevention and support strategies by the university. Finally, the overall response rate was satisfactory and the different academic groups were homogeneously represented. To improve knowledge and awareness of the burden of mental illness among academics, this study would need to be expanded on a national scale.

## Conclusions

High external pressure, commonly referred to as the “publish or perish” aphorism, may play an important role in the mental health of academics. The higher prevalence of depressive and anxiety symptoms among junior academics may be an impact of career uncertainty and experiences in the demanding academic environment on the mental health of the younger ones. Because mental health problems are in turn associated with poor academic outcomes and productivity [48, 49] as well as retention in academic careers, investing in providing coping tools for junior academics could be strategic for both personal and professional empowerment. Universities should provide greater support to early career academics to encourage their effective and healthy contribution to the advancement of research. Nonetheless, strategies aimed at overcoming career uncertainty and supporting the process of fundraising for research would certainly contribute positively to this public health problem.

## Data Availability

The dataset generated and analysed during the current study are not publicly available due permission not requested during study protocol submission and to participants during data collection but could be available from the corresponding author on reasonable request and after amendment of the study protocol.

## Abbreviations

UN-SAD: Symptoms of Anxiety and Depression within the UNiversity community
PHQ-9: Patient Health Questionnaire–9
GAD-7: General Anxiety Disorder–7
